# Increased compliance with tumor treating fields therapy is prognostic for improved survival in the treatment of glioblastoma: a subgroup analysis of the EF-14 phase III trial

**DOI:** 10.1007/s11060-018-03057-z

**Published:** 2018-12-01

**Authors:** S. A. Toms, C. Y. Kim, G. Nicholas, Z. Ram

**Affiliations:** 1 Department of Neurosurgery, Warren Alpert Medical School of Brown University, Providence, RI USA; 2 Seoul National University, Bundang, South Korea; 3 Ottawa Hospital Research Institute, Ottawa, ON Canada; 4 Tel Aviv Medical Center, Tel Aviv, Israel

**Keywords:** Glioblastoma, Tumor treating fields, Compliance, Monthly usage

## Abstract

**Background:**

Tumor treating fields (TTFields) is a non-invasive, antimitotic therapy. In the EF-14 phase 3 trial in newly diagnosed glioblastoma, TTFields plus temozolomide (TTFields/TMZ) improved progression free (PFS) and overall survival (OS) versus TMZ alone. Previous data indicate a ≥ 75% daily compliance improves outcomes. We analyzed compliance data from TTFields/TMZ patients in the EF-14 study to correlate TTFields compliance with PFS and OS and identify potential lower boundary for compliance with improved clinical outcomes.

**Methods:**

Compliance was assessed by usage data from the NovoTTF-100A device and calculated as percentage per month of TTFields delivery. TTFields/TMZ patients were segregated into subgroups by percent monthly compliance. A Cox proportional hazard model controlled for sex, extent of resection, *MGMT* methylation status, age, region, and performance status was used to investigate the effect of compliance on PFS and OS.

**Results:**

A threshold value of 50% compliance with TTFields/TMZ improved PFS (HR 0.70, 95% CI 0.47–1.05) and OS (HR 0.67, 95% CI 0.45–0.99) versus TMZ alone with improved outcome as compliance increased. At compliance > 90%, median survival was 24.9 months (28.7 months from diagnosis) and 5-year survival rate was 29.3%. Compliance was independent of gender, extent of resection, *MGMT* methylation status, age, region and performance status (HR 0.78; p = 0.031; OS at compliance ≥ 75% vs. < 75%).

**Conclusion:**

A compliance threshold of 50% with TTFields/TMZ correlated with significantly improved OS and PFS versus TMZ alone. Patients with compliance > 90% showed extended median and 5-year survival rates. Increased compliance with TTFields therapy is independently prognostic for improved survival in glioblastoma.

**Electronic supplementary material:**

The online version of this article (10.1007/s11060-018-03057-z) contains supplementary material, which is available to authorized users.

## Introduction

Glioblastoma (GBM) is the most common and aggressive adult brain tumor, accounting for 56% of all gliomas and 15% of all primary brain tumors with an annual incidence in the United States that increases with age—ranging from 0.2 per 100,000 in 0–19 year old population to the highest rate of 15.3 per 100,000 in the 75–84 year old population [1]. Glioblastoma remains incurable with a median survival of only 15 months until recently [2]. The previous standard treatments for newly diagnosed GBM include maximally safe surgical resection followed by radiation therapy (RT) and adjuvant temozolomide (TMZ) chemotherapy [3].

Tumor treating fields (TTFields) are a unique treatment modality [4, 5] for GBM that affects rapidly dividing glioma cells through the action of low-intensity, intermediate frequency (200 kHz) alternating electric fields [6–9] that act on microtubules and septin fibers of proliferating cancer cells to disrupt mitosis, inducing mitotic cell death, mitotic catastrophe, and cellular stress characterized by autophagy, and endoplasmic reticulum stress [6–13]. TTFields inhibit DNA damage repair by the expression of DNA repair genes in the BRCA1 pathway [14] and impair cellular migration and invasion [15]. TTFields increases cell death when combined with anti-PD1, chemotherapy and radiotherapy [16–19].

The phase 3 EF-11 study of TTFields in recurrent GBM demonstrated comparable efficacy to best physician choice chemotherapy without treatment limiting systemic adverse effects [20]. Post hoc analysis of the EF-11 trial data showed significantly longer median OS with TTFields at compliance rate of ≥ 75% (≥ 18 h daily) versus those with a < 75% compliance rates [21] and high compliance rates of > 90% with EF-11 responders [22]. The Patient Registry Dataset (PRiDe) showed significant improvement in median OS at daily compliance rates of ≥ 75% versus < 75% [23]. The phase 3 EF-14 study in newly diagnosed GBM demonstrated that TTFields plus maintenance TMZ therapy significantly improved PFS and OS without decline in health related quality of life (HRQOL) versus TMZ alone [24–26]. The National Comprehensive Cancer Network (NCCN) has recently (2018) recommended TTFields with TMZ as a standard Category 1 treatment option for newly diagnosed GBM [27].

Unlike systemic therapies, TTFields are only active against cancer cells while the transducer arrays are placed on the scalp and the field generator is continuously administering alternating electric fields of a specific intensity (200 KHz) for GBM. There are no peak-trough fluctuations or half-life associated with TTFields. The specificity of TTFields on anti-mitotic activity of rapidly dividing glioma cells, while sparing normal cell division, enables near continuous cancer therapy with minimal systemic adverse effects. Therefore, active compliance with TTFields therapy is a critical parameter for clinical benefit.

The objective of this subgroup analysis of the EF-14 phase III trial data was to analyze compliance data to correlate TTFields compliance with PFS and OS and identify potential lower boundary for compliance rates with improved clinical outcomes.

## Methods

This subgroup analysis is based on TTFields plus TMZ and TMZ alone patient data from the EF-14 study [24]. The EF-14 trial was a randomized, open-label trial, which enrolled 695 newly diagnosed patients with GBM whose tumor was either resected or biopsied and had also completed concomitant radiation therapy with adjuvant TMZ therapy. Patients were randomized 2:1 to TTFields plus maintenance TMZ chemotherapy (n = 466) or temozolomide alone (n = 229). Temozolomide was administered to both groups (150–200 mg/m^2^) for 5 days per 28-day cycle (6–12 cycles). The median time from diagnosis to randomization in both groups was 3.8 months [24].

The primary outcome of this subgroup analysis was to assess the percentage of monthly TTFields compliance as an independent predictor of PFS and OS compared with patients in the TMZ alone treatment group. Compliance data are derived from the internal computerized log file of each NovoTTF-100A (Optune^®^) device. Percent of the total treatment time during which the NovoTTF-100A treated patients actually received treatment was calculated by analyzing the log file of each device and dividing the total device ‘ON’ time by the prescribed number of 1 month treatment courses.

Patient compliance was calculated as the average percentage of each month the system was delivering TTFields. Progression-free survival and OS data from the TTFields plus TMZ treated group were analyzed in subgroups based on monthly compliance levels of < 75% or ≥ 75% and finer monthly compliance bins of 0 to ≤ 30%, 30% to ≤ 50%, 50% to ≤ 60%, 60% to ≤ 70%, 70% to ≤ 80%, 80% to ≤ 90%, 90% to ≤ 100%.

The PFS and OS survival curves were constructed using the Kaplan–Meier method. Cox proportional hazards model was used to analyze treatment compliance as an independent predictor of survival controlling for treatment group, sex, *MGMT* methylation status, resection status, Karnofsky Performance Status (KPS) and country of residence (United States versus outside the United States). The threshold for significant interactions in the model was specified at an α of 0.05.

## Results

In the EF-14 study, 466 patients were randomized to the TTFields plus TMZ therapy group and 229 were randomized to the TMZ alone group [24]. The patient disposition is shown in Supplementary Fig. 1. In summary, for the TTFields plus TMZ group—the majority of patients were men (68%) with a median age of 56 years, and a KPS of 90% [24]. The *MGMT* promoter region was unmethylated in 54% and methylated in 36% patients in the TTFields plus TMZ group [24]. Table 1 shows the baseline demographic characteristics of the TTFields plus TMZ group separated into subgroups based on percent compliance. Overall, the separate percent compliance groups were matched in baseline characteristics both with each other and the full data set of the primary study.

**Table 1:** Baseline demographics by TTFields percent average daily compliance

% Compliance	0 to ≤ 30 (n = 22)	30 to ≤ 50 (n = 40)	50 to ≤ 60 (n = 42)	60 to ≤ 70 (n = 46)	70 to ≤ 80 (n = 91)	80 to ≤ 90 (n = 166)	90 to ≤ 100 (n = 43)	TMZ alone (n = 229)
Median age, years (range)	55.5 (30–70)	57.5 (25–78)	54.5 (22–79)	55.0 (20–83)	56.0 (30–78)	56.0 (28–80)	52.0 (19–68)	57.0 (19–80)
KPS, median (range)	80.0 (70–100)	90.0 (70–100)	90.0 (70–100)	90.0 (60–100)	90.0 (70–100)	90.0 (70–100)	90.0 (70–100)	90.0 (70–100)
Extent of resection, n (%)								
Biopsy only	6 (27)	4 (10)	2 (5)	8 (17)	10 (11)	23 (14)	5 (12)	29 (13)
Partial/complete	5 (23)	14 (35)	18 (43)	15 (33)	34 (37)	52 (31)	11 (26)	77 (34)
Gross total resection	11 (50)	22 (55)	22 (52)	23 (50)	47 (52)	91 (55)	27 (63)	123 (54)
MGMT tissue available and tested, n (%)	16 (73)	34 (85)	39 (93)	35 (76)	71 (78)	142 (86)	37 (86)	185 (81)
Methylated	5 (31.3)	14 (41.2)	12 (30.8)	13 (37.1)	24 (33.8)	49 (34.5)	15 (40.5)	77 (41.6)
Unmethylated	10 (62.5)	15 (44.1)	24 (61.5)	20 (57.1)	41 (57.7)	76 (53.5)	17 (45.9)	95 (51.4)
Invalid	1 (6.3)	5 (14.7)	3 (7.7)	2 (5.7)	6 (8.5)	17 (12.0)	5 (13.5)	13 (7.0)

**Fig. 1:**
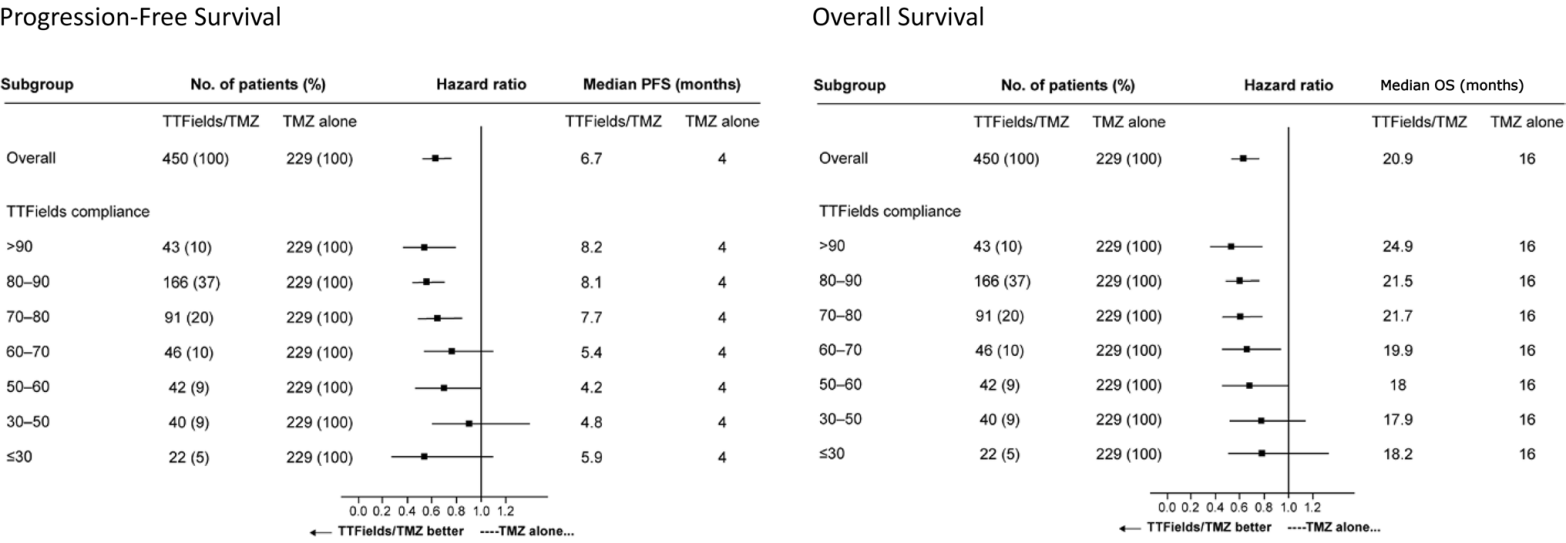
Forest plots show the effect of treatment compliance with TTFields plus TMZ on PFS and OS. A threshold value of 50% compliance with TTFields plus TMZ was needed to show a significant extension of OS compared to TMZ alone. Both PFS and OS were extended with treatment compliance levels > 50%. A trend in favor of longer PFS and OS was seen with higher rates of treatment compliance

Analysis of the more refined rates of compliance (smaller bin sizes) shows a trend in favor of longer PFS and OS with progressively higher levels of monthly compliance. A threshold value of ≥ 50% average monthly compliance with TTFields plus TMZ (Fig. 1) was needed to show an extension of PFS (HR 0.70, 95% CI 0.47–1.05) and OS (HR 0.67, 95% CI 0.45–0.99) compared to TMZ alone. Both PFS and OS were extended when compliance was increased beyond 50%, indicating progressively increased gains in PFS and OS as compliance increases.

**Fig. 2:**
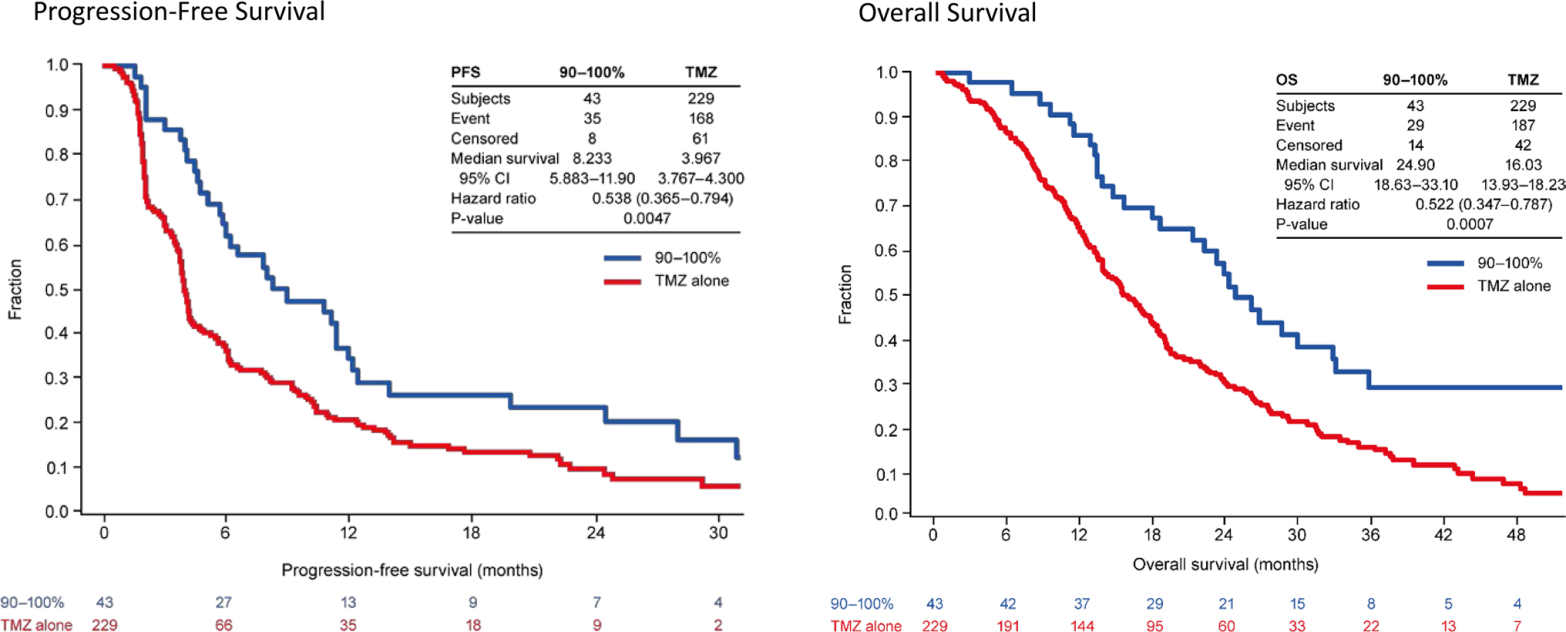
Newly diagnosed GBM patients had maximal treatment benefit from TTFields plus TMZ with compliance rates > 90% with a median overall survival of 24.9 months (28.7 months from diagnosis)

**Fig. 3:**
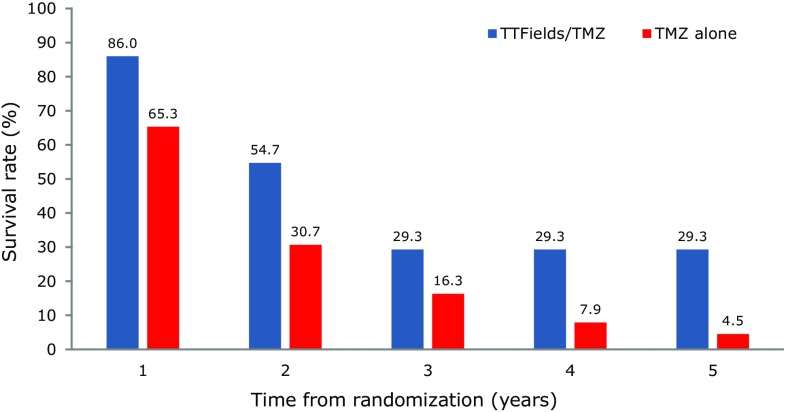
The annual survival rate was highest for newly diagnosed GBM patients with compliance rates > 90% with a 29.3% survival rate over 5 years from randomization

Patients with TTFields plus TMZ compliance levels of > 90% showed maximum survival benefits (Fig. 2), with a median PFS of 8.2 months for the TTFields plus TMZ group compared to 4.0 months in the TMZ alone group (HR 0.538, 95% CI 0.365–0.794; p = 0.0047) and an OS of 24.9 months (28.7 months from diagnosis since time from diagnosis to randomization was 3.8) in the TTFields plus TMZ arm compared to 16.0 months in the TMZ alone group respectively (HR 0.522, 95% CI 0.347–0.787; p = 0.0007). TTFields plus TMZ treated patients with > 90% compliance rate had a 5-year survival rate of 29.3% (Fig. 3).

A compliance level of ≥ 75% monthly duration of treatment with TTFields plus TMZ was an independent predictor of OS, as was methylated *MGMT* status, age and KPS (Table 2) regardless of treatment arm (reference values—compliance < 75%), sex (male), resection (biopsy), *MGMT* (negative), and region (USA).

**Table 2:** Cox proportional hazards model for OS in TTFields/TMZ patients

Parameter	Parameter value	Hazard ratio	Two-sided p-value
Treatment arm	Compliance ≥ 75%	0.781	0.031
Sex	Female	0.800	0.069
Resection status	Gross total resection	0.789	0.202
	Partial resection	0.777	0.181
MGMT status	Methylated	0.510	< 0.001
	Unknown	0.810	0.131
Region	Outside the USA	1.157	0.199
Age		1.021	< 0.001
KPS		0.984	0.006

## Discussion

In this subgroup analysis of EF-14 study patients receiving TTFields plus TMZ treatment, a threshold value of ≥ 50% average compliance with TTFields plus TMZ showed an extension of PFS and OS compared to TMZ alone. Further, patients with monthly compliance > 90% had maximal survival benefit with a median survival of 24.9 months (28.7 months from diagnosis) and a 5-year survival of 29.3%. This effect was independent of other prognostic factors such as performance status, age, and *MGMT* methylation status. Compliance was an independent predictor of OS in the full 5-year dataset (≥ 75% vs. < 75%) [24].

Post hoc analysis of the EF-11 trial data demonstrated longer median OS in TTFields treated recurrent GBM patients with a compliance rate of ≥ 75% compared to those with a < 75% compliance rate (7.7 vs. 4.5 months; p = 0.042) [21]. This early analysis supported a preliminary target level for treatment compliance (≥ 75%) in clinical practice as well as evidence for a trend suggesting that higher levels of survival benefit were associated with increasing compliance [21]. Data from the PRiDe registry—using data from real-world recurrent GBM patients—also demonstrated improved OS with daily compliance rates ≥ 75% [23]. The results of the EF-14 sub-group analysis further support a threshold compliance rate of ≥ 75% for a survival benefit when compared to a compliance rate of < 75% in newly diagnosed GBM patients treated with TTFields plus TMZ. This study demonstrates that a minimal compliance threshold of > 50% with TTFields plus TMZ treatment correlated with significantly improved survival outcomes compared to TMZ alone for newly diagnosed GBM. TTFields were administered to GBM patients with recurrent disease as monotherapy in the EF-11 study and as combination therapy with TMZ in newly diagnosed GBM patients in the EF-14 study. The earlier disease stage and combined treatment may account for the survival benefits seen at a lower minimal compliance threshold in this subgroup analysis of the EF-14 study.

A variety of social and clinical factors contribute to patient compliance with TTFields therapy. Though TTFields are non-invasive and the Optune device is designed to preserve patient functioning during daily activities, initiating TTFields therapy requires some lifestyle modifications when compared to RT or systemic therapies. Some patients may be reluctant to comply with the head shaving required with every array change and wearing the arrays on a shaved head may make some patients self-consciousness, calling attention to their condition [28]. Healthcare providers experienced with TTFields therapy can provide patients assistance with incorporating the therapy in their daily life [28].

TTFields, like oral cancer treatment regimens, are administered in the home and outpatient setting and places the burden of compliance on the patient and their caregivers. Patient, healthcare provider, and treatment related factors can contribute to improved adherence or compliance with oral cancer therapy regimens [29]. Patient related factors include physical limitations, psychological, and social issues such as religious or cultural factors and the lack of a support system. The healthcare provider can also negatively influence compliance with therapy through poor communication and relationship with the patient, as well as failing to optimally select appropriate patients for oral cancer therapy regimens [29].

A good home support system is critical when considering TTFields therapy for a GBM patient [28]. A patient should have at least one support person who can assist with the Optune device operation, assist with managing adverse events, scalp maintenance and array placement. Patients with cognitive issues or poor performance status have been suggested to be more likely to be less compliant with TTFields treatment without home support [28]. However, the current study showed compliance to be independent of KPS and age as a predictor of PFS and OS, contradicting this suggestion. Treatment-related factors influencing compliance include complex treatment regimens, concomitant treatments and side effects. TTFields are not associated with systemic side effects and are less likely to affect concomitant systemic therapy.

The most common side effect in clinical trials was skin irritation for patients treated with TTFields [20, 24, 25]. Dermatological adverse events were the most common adverse events associated with TTFields; 52% of TTFields plus TMZ patients in the EF-14 trial reported mild to moderate skin irritation [24]. Skin irritation is due primarily to the nearly continuous contact of the transducer arrays with the patients shaved scalp between array changes. These events include allergic and irritant dermatitis, mechanical lesions, ulcers and skin lesions [30]. However, most of these dermatological AEs can either be prevented with proper shaving techniques, skin care and array relocations, or treated with appropriate topical regimens as required [30]. Effective skin care strategies can maximize compliance with TTFields therapy and maintain patient QoL over the course of treatment.

A limitation of this study is that it is based on a subgroup analysis of the phase 3 EF-14 trial, and inherently subgroup analyses are prone to type I errors limiting the veracity of the results [31]. In this instance, the subgroup analysis was prespecified in the protocol. However, the results of this investigation corroborate the results of similar analyses of prior clinical investigations [21–23].

## Conclusions

In this subset analysis of the EF-14 trial, a compliance threshold of 50% with TTFields plus TMZ treatment correlated with significantly improved outcomes compared to TMZ alone. Higher levels of treatment compliance with TTFields plus TMZ were associated with increased durations of progression free- and overall-survival suggesting a dose response mechanism for TTFields. This effect was independent of other prognostic factors such as performance status, age, and *MGMT* methylation status. Patients with compliance over 90% had a median survival of 24.9 months (28.7 months from diagnosis) and a 3-, 4-, and 5-year survival of 29.3%. This plateau effect on long term survival has been identified in other GBM treatments which have known immunologic mechanisms of action [32, 33]. The importance of compliance with TTFields therapy in real world clinical settings should be strongly conveyed to patients by their treating physicians and other allied healthcare providers.

## Electronic supplementary material

Below is the link to the electronic supplementary material.

Supplementary Figure 1 Patient disposition in the 5-year final analysis of the EF-14 Study [24]. (PPTX 41 KB)
