# Supplementary material for: Increased compliance with tumor treating fields therapy is prognostic for improved survival in the treatment of glioblastoma: a subgroup analysis of the EF-14 phase III trial
Source: J Neurooncol. 2018 Dec 1;141(2):467–73. doi: 10.1007/s11060-018-03057-z (PMC6342854; doi:10.1007/s11060-018-03057-z)
Supplement: Supplementary file 1 — Supplementary Figure 1 Patient disposition in the 5-year final analysis of the EF-14 Study [24]. (PPTX 41 KB) [file 11060_2018_3057_MOESM1_ESM.pptx]

## Slide 1
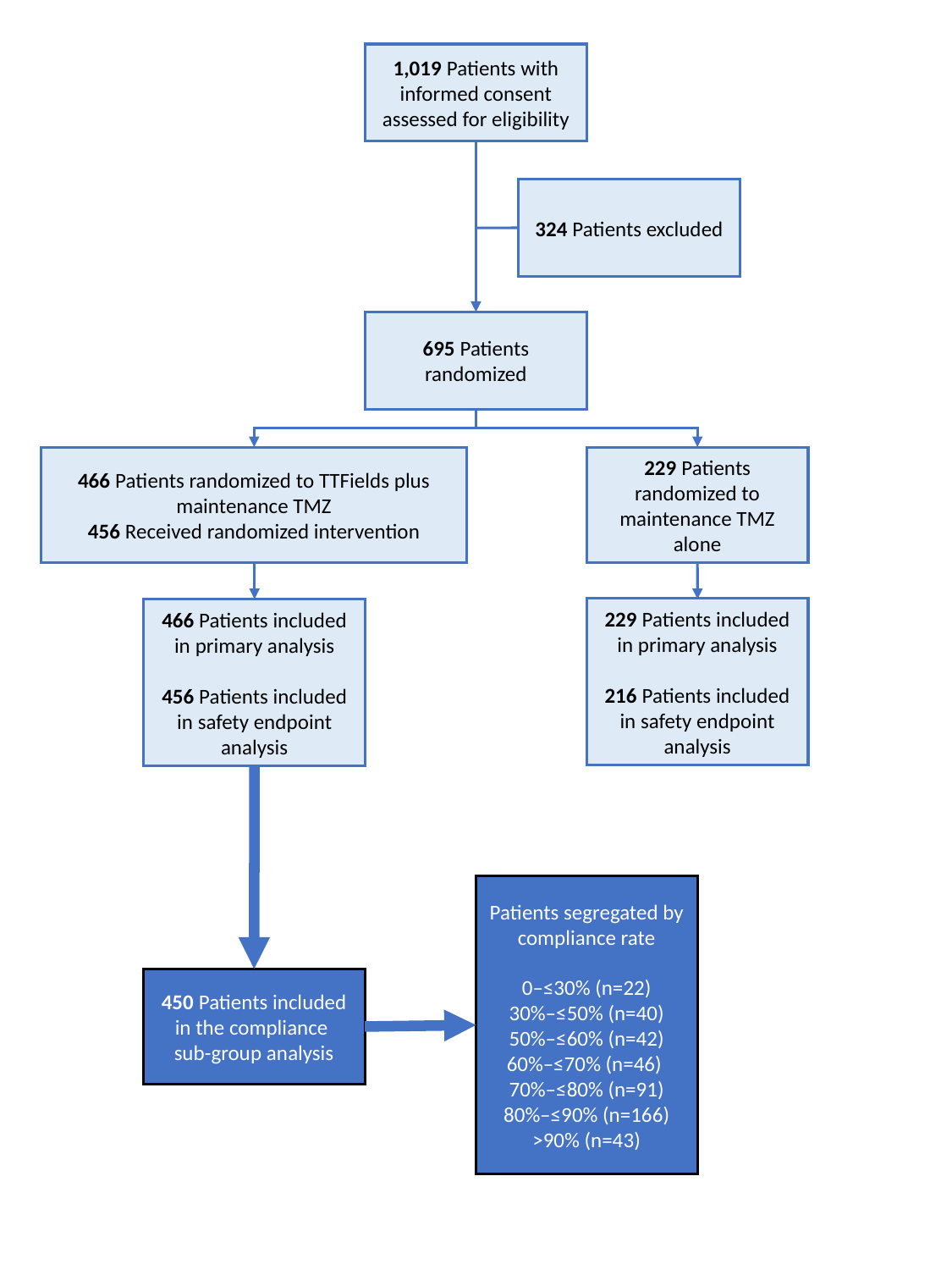

1,019 Patients with informed consent assessed for eligibility
324 Patients excluded
695 Patients randomized
466 Patients randomized to TTFields plus maintenance TMZ
456 Received randomized intervention
229 Patients randomized to maintenance TMZ alone
229 Patients included in primary analysis
216 Patients included in safety endpoint analysis
466 Patients included in primary analysis
456 Patients included in safety endpoint analysis
Patients segregated by compliance rate
0–≤30% (n=22)
30%–≤50% (n=40)
50%–≤60% (n=42)
60%–≤70% (n=46)
70%–≤80% (n=91)
80%–≤90% (n=166)
>90% (n=43)
450 Patients included in the compliance
sub-group analysis
